# Optimal time period for blood glucose level evaluation after total knee arthroplasty in patients without diabetes: a prospective, observational study

**DOI:** 10.1186/s13018-022-03023-6

**Published:** 2022-02-24

**Authors:** Masaki Nagashima, Kenichiro Takeshima, Ryo Sasaki, Noriyuki Aibara, Shuji Aomatsu, Toshiro Otani, Ken Ishii

**Affiliations:** 1 Department of Orthopaedic Surgery, School of Medicine, International University of Health and Welfare, 4-3, Kōzunomori, Narita City, Chiba 286-8686 Japan; 2 Department of Orthopaedic Surgery, International University of Health and Welfare Mita Hospital, 1-4-3, Mita, Minato-ku, Tokyo 108-8329 Japan; 3 Department of Orthopaedic Surgery, International University of Health and Welfare Narita Hospital, 852, Hatakeda, Narita City, Chiba 286-8520 Japan; 4 Department of Orthopaedic Surgery, International University of Health and Welfare Ichikawa Hospital, 6-1-14, Konodai, Ichikawa City, Chiba 272-0827 Japan

**Keywords:** Total knee arthroplasty, Periprosthetic infection, Blood glucose level, Diabetes mellitus

## Abstract

**Background:**

Postoperative hyperglycemia has been reported to be a risk factor for postoperative infection even in patients without diabetes mellitus (DM). However, there is no standard for how long blood glucose level (BGL) monitoring should be performed after total knee arthroplasty (TKA). The purpose of this study was to determine the optimal time period for BGL evaluation after TKA in patients without DM.

**Methods:**

This prospective study included 132 knees of 110 patients who underwent TKA between March 2018 and July 2021 in our hospital. Fasting BGLs were measured preoperatively, at 9:00 PM on the day of surgery (DOS), and at 7:00 AM on postoperative days (PODs) 1, 2, and 3. Patients were divided into two groups with a preoperative hemoglobin A1c (HbA1c) cut-off value of 5.9%, and the BGLs on POD 1 were compared between the two groups.

**Results:**

The BGLs were significantly higher on the DOS, POD 1, and POD 2 than preoperative levels. The BGL was significantly higher on POD 1 than at any other time point. Patients with an HbA1c ≥ 5.9% had significantly higher BGLs than those with an HbA1c < 5.9% on POD 1.

**Conclusions:**

The optimal time period for BGL evaluation after TKA in patients without DM was considered to be from postoperative to POD 2. Patients with an HbA1c ≥ 5.9% may require careful perioperative glycemic control.

## Background

Periprosthetic infection is one of the serious complications after total knee arthroplasty (TKA). The infection rate has been reported to be gradually increasing [1] and is the major cause of revision TKA in recent years [2]. Once the infection occurs, it can lead to additional surgeries, prolonged treatment periods, and increased financial costs [3]. Periprosthetic infection has an impact not only on the patient, but also on the medical staff. Since postoperative hyperglycemia caused by surgical stress has been reported to be a risk factor for periprosthetic infection even in patients without diabetes mellitus (DM) [4–7], strict perioperative glycemic control is important for patients with and without DM. The Center for Disease Control and Prevention (CDC) provided guidelines that strongly recommended that the blood glucose level (BGL) should be controlled to less than 200 mg/dl in patients with and without DM [8]. In addition, American College of Surgeons (ACS) and Surgical Infection Society (SIS) Surgical Site Infection guidelines, 2016 Update mentioned that target perioperative BGL should be from 110 to 150 mg/dl in all patients, regardless of diabetic status [9]. However, the duration of glycemic control was not identified in both guidelines [8, 9]. Because uncontrolled DM is reported to be a risk factor for postoperative infection [10], patients with DM are subjected to strict glycemic control. However, in patients without DM, glycemic control is generally not performed, and it is unclear how BGLs change after TKA. The same strict glycemic control for non-diabetic patients as for diabetic patients will increase the burden on patients, as well as on the medical staff. For non-diabetic patients, although there is a need for a standard for how long blood glucose monitoring should be performed after TKA, the details are unclear. The purpose of this study was to investigate perioperative BGLs in patients without DM undergoing unilateral primary TKA and to determine the optimal time period for BGL evaluation after TKA.

## Methods

### Patients

This was a prospective, observational study. Between March 2018 and July 2021, 160 primary unilateral TKAs in 136 patients with osteoarthritis were performed consecutively in our hospital and included in this study. The exclusion criteria were as follows: (1) patients with DM or hemoglobin A1c (HbA1c) greater than 6.5% at preoperative examination (*n* = 23); (2) patients needed oral corticosteroid treatment over the past 6 months (*n* = 1); (3) TKAs that needed stem extension of the femoral component (*n* = 2); or (4) patients required other treatment within the first week after the TKA (*n* = 2). Thus, 132 knees of 110 patients were enrolled in this study. Sex, age, body mass index (BMI), preoperative HbA1c, and the preoperative femorotibial angle of the patients were recorded (Table 1).

**Table 1:** Preoperative demographics of the patients

	*n* = 132
Sex	
Female	104
Male	28
Age (y)	74.9 ± 6.6
BMI (kg/m^2^)	25.2 ± 4.2
HbA1c (%)	5.7 ± 0.3
Pre-op FTA (°)	182.6 ± 9.5

*BMI* body mass index, *HbA1c* hemoglobin A1c, *FTA* femorotibial angle

### Operative procedure

All patients were operated on under general anesthesia and femoral and sciatic nerve blocks (0.375% ropivacaine 20 ml and 15 ml were used, respectively). None of the patients received intravenous glucocorticoids for vomiting prophylaxis. TKAs were performed with tourniquets, using the measured resection technique aiming for neutral alignment of the knee. The medial parapatellar approach was used. Distal femoral cutting was performed at a valgus angle of 5–7 degrees with an intramedullary alignment guide. Rotation of the femoral component was 3–7 degrees external rotation from the posterior condylar axis, aiming at the surgical epicondylar axis. Proximal tibial cutting was performed with an extramedullary guide. Rotation of the tibial component was indexed to the Akagi line [11]. In all patients, the patella was resurfaced, and the thickness of bone resection was that of the patellar component to be placed. Lateral patellar facetectomy was performed by a bone saw for the lateral aspect of the patella that was not covered by the implant to avoid lateral patellar facet impingement [12, 13]. All components were fixed with bone cement. The Persona (Zimmer Biomet, Warsaw, IN, USA) was used in 76 knees, TriMax (Ortho Development, Draper, UT, USA) was used in 30 knees, Attune (Depuy, Warsaw, IN, USA) was used in 24 knees, and ACTIYAS Total Knee System (Kyocera, Kyoto, Japan) was used in 2 knees. All TKAs were fixed-bearing posterior stabilizing prostheses.

### Postoperative care

Oral hydration was allowed 4 h after surgery. A 1000 ml glucose free acetated Ringer's solution was given intravenously during 12 h postoperatively. Full-weight walking and range of motion exercises were started the day after surgery in accordance with pain tolerance. For postoperative pain control, a continuous femoral nerve block delivering 0.1% ropivacaine, 4 ml per hour, was used for 24 h, and celecoxib, 200 mg twice daily, was administered orally for two weeks after TKA. If pain control was inadequate, a diclofenac sodium suppository (50 mg) and/or intravenous acetaminophen (1000 mg) was used, as appropriate. To prevent deep vein thrombosis, although no patients received pharmacological prophylaxis, intermittent pneumatic compressions for both feet were used for one day, and compression stockings were used until the patient was able to walk. For antibiotic dosing, cefazolin or clindamycin was used, and 1 g of each was administered preoperatively and every 8 h up to 48 h after the TKA.

### Measurement of BGL

Fasting BGLs were measured in this study. Until September 2019, BGL measurements were performed preoperatively, at 9:00 PM on the day of surgery (DOS), and at 7:00 AM on postoperative days (PODs) 1 and 2. After October 2019, BGL measurement on POD 3 was performed instead of on POD 2; BGL measurements were performed on PODs 2 and 3 in 73 and 59 knees, respectively.

### Statistical analysis

One-way analysis of variance was used to compare the BGLs at each measurement time point. Multiple regression analysis was used to evaluate the risk factors for hyperglycemia on POD 1. In the analysis, preoperative HbA1c, operative time, age, BMI, sex, and preoperative BGL were included as the potential risk factors. When hyperglycemia was defined as BGL exceeding 150 mg/dl, as described in the ACS and SIS guidelines [9], the cut-off value of preoperative HbA1c to predict hyperglycemia on POD 1 was examined using the receiver operating characteristics (ROC) curve. The point nearest to the top-left most corner of the ROC curve was chosen as the cutoff value. The patients were divided into two groups with the HbA1c cut-off value, and the BGLs and incidence of patients with BGLs that exceeded 150 mg/dl on POD 1 were compared between the two groups, using Student’s *t*-test and the *χ*^2^ test. A *P* value of < 0.05 was considered significant. All statistical analyses were performed with BellCurve for Excel ver. 3.21 (Social Survey Research Information, Tokyo, Japan).

## Results

**Fig. 1:**
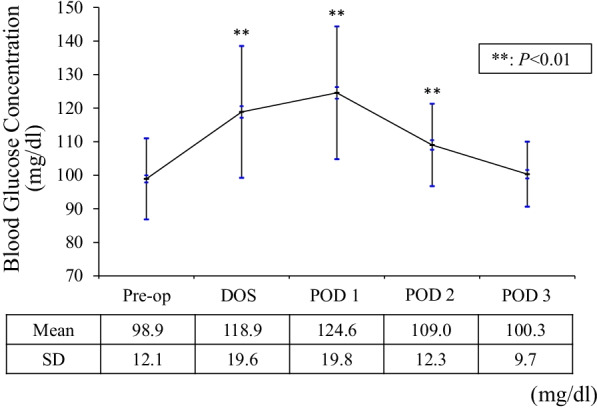
Perioperative blood glucose level (BGL) changes. The mean BGLs are significantly higher on the day of surgery, postoperative day (POD) 1, and POD 2 than preoperative levels. The highest BGL is found on POD 1. *SD* standard deviation

The mean operative time was 97.8 ± 14.1 min, and there were no patients who required blood transfusion postoperatively. Perioperative BGL changes are shown in Fig. 1. The mean BGLs were significantly higher on the DOS, POD 1, and POD 2 than preoperative levels (*P* < 0.001). The highest mean BGL was found on POD 1, and it was significantly higher than at any other time point (*P* = 0.036 for DOS and *P* < 0.001 for the others). The mean BGL on the DOS was significantly higher than on PODs 2 and 3 (*P* < 0.001). The mean BGL was significantly higher on POD 2 than on POD 3 (*P* = 0.018). There were no cases of BGLs exceeding 200 mg/dl at any time point, but only on the DOS and POD 1, BGLs exceeded 150 mg/dl in 7 (5.3%) (range: 156–188 mg/dl) and 14 (10.6%) (range: 151–198 mg/dl) cases, respectively.

**Fig. 2:**
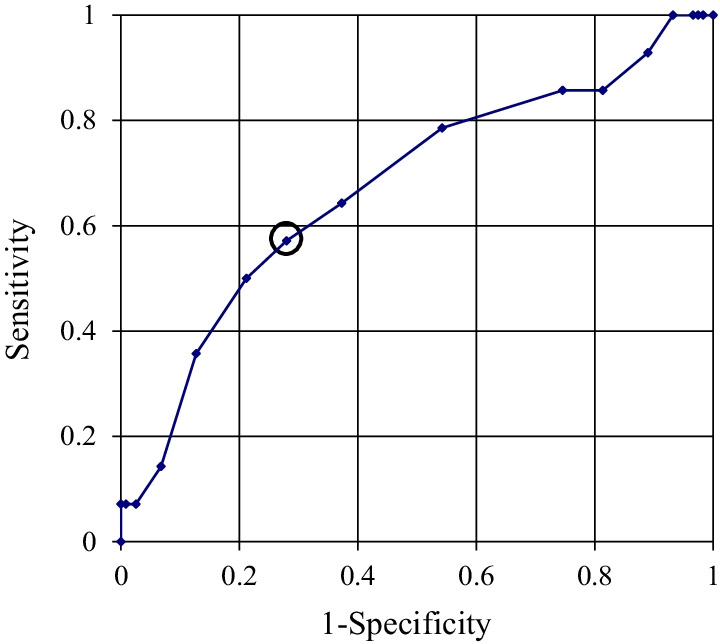
ROC curve for preoperative HbA1c in the prediction of BGL of > 150 mg on POD 1. The cut-off value for preoperative HbA1c for prediction of hyperglycemia above 150 mg/dl on POD 1 was chosen to be 5.9% (black circle)

An increased BGL on POD 1 was significantly related to a high preoperative HbA1c (Table 2). On the ROC curve, the cut-off value for preoperative HbA1c for prediction of hyperglycemia above 150 mg/dl on POD 1 was chosen to be 5.9%, and the area under ROC curve was 0.673 (*P* = 0.036), and a sensitivity and specificity were 57.1% and 72.0%, respectively (Fig. 2). Patients with an HbA1c of 5.9% or more had significantly higher BGLs on POD 1 than patients with an HbA1c of less than 5.9% (*P* = 0.002). And in patients with an HbA1c of 5.9% or more, the incidence of patients with BGL exceeding 150 mg/dl on POD 1 was 19.5%, significantly higher than the 6.6% in patients with an HbA1c of less than 5.9% (*P* = 0.026) (Table 3). No cases of infection were observed within 3 months after the TKA.

**Table 2:** Multiple regression analysis of the relationship between the BGL on POD 1 and potential risk factors

Risk factor	Partial regression coefficient	95% confidence interval	*P* value
Pre-op HbA1c (%)	13.60	1.72 to 25.47	0.025
BMI (kg/m^2^)	− 0.39	− 1.30 to 0.52	0.398
Pre-op BGL (mg/dl)	0.11	− 0.19 to 0.42	0.465
Operative time (minutes)	0.08	− 0.18 to 0.34	0.561
Age (y)	0.12	− 0.45 to 0.68	0.688
Sex (male)	1.42	− 7.40 to 10.25	0.750

*HbA1c* hemoglobin A1c, *BMI* body mass index, *BGL* blood glucose level

**Table 3:** Comparison of the BGLs and number of patients with a BGL above 150 mg/dl on POD 1 with the HgA1c cut-off value of 5.9%

	HbA1c < 5.9% (*n* = 91)	HbA1c ≥ 5.9% (*n* = 41)	*P* value
POD 1 BGL (mg/dl)	120.9 ± 18.4	132.6 ± 20.5	0.002
POD 1 BGL			
< 150 mg/dl (n)	85	33	0.026
≥ 150 mg/dl (n)	6	8	

*HbA1c* hemoglobin A1c, *BGL* blood glucose level

## Discussion

The postoperative BGLs in patients without DM after TKA were highest on POD 1 and then decreased. Postoperative BGLs were significantly higher up to POD 2 compared to the preoperative levels. Preoperative HbA1c was significantly associated with high BGLs on POD 1.

Even in patients without DM, postoperative hyperglycemia is reported to be a risk factor for postoperative infection in total joint arthroplasty [4–7]. One of the reasons reported was that hyperglycemia affected all major components of innate immunity and reduced neutrophil activity [14]. There have been several reports on the association between hyperglycemia and infection after total joint arthroplasty. Mraovic et al. measured postoperative fasting BGL and reported a three-fold increase in infection rates in non-diabetic patients with a fasting BGL of 140 mg/dl or more on POD 1 [4]. Maradit et al. reported a significantly increased infection rate at BGLs above 180 mg/dl at one week before and after surgery [15]. Perioperative hyperglycemia has been reported to be associated with various complications, such as urinary tract infection, ileus, and aseptic loosening, as well as infection [10, 16]. These reports varied in the timing of BGL evaluations whether fasting or random BGL was used, but these results suggested that adequate glycemic control was necessary even in patients without DM.

Several guidelines also indicate the necessity for glycemic control [8, 9]. The CDC guidelines strongly recommended that the BGL should be controlled to less than 200 mg/dl in patients with and without DM [8]. The ACS and SIS guidelines mentioned that target perioperative BGL should be from 110 to 150 mg/dl in all patients, regardless of diabetic status [9]. However, the timing of BGL evaluation was not specified in either set of guidelines. Providing non-diabetic patients with the same level of glycemic control as diabetic patients can be a significant burden for both patients and healthcare professionals. Thus, perioperative BGLs in patients without DM undergoing primary TKA were investigated to determine the optimal time period for blood glucose evaluation in the present study. Postoperative BGLs were significantly higher up to POD 2 compared to preoperative levels, suggesting that the optimal time period for BGL evaluation in patients without DM was from postoperative to the morning of POD 2. If hyperglycemia is observed during that period, prompt glycemic control should be initiated.

The postoperative BGLs in patients without DM after TKA were highest on POD 1 in the present study. The development of stress hyperglycemia is caused by transient insulin resistance and a highly complex interplay of counter-regulatory hormones such as catecholamines, growth hormone, cortisol, and cytokines [17, 18]. Since the hyperglycemia occurs as a result of the complex interplay, we believe that the peak of hyperglycemia may have been POD 1 rather than immediately after surgery. In fact, it has been reported that postoperative reduction in insulin sensitivity was most pronounced on POD 1 [19]. Varady et al. examined the timing of postoperative blood glucose monitoring after total joint arthroplasty and stated that the BGL was highest at 9 PM on the DOS, and, therefore, this was the optimal time to detect hyperglycemia. In contrast, even on the morning after surgery, more than 20% of patients had a BGL of 137 mg/dl or more [20].

In the present study, increased preoperative HbA1c was significantly associated with hyperglycemia on POD 1. Since this association alone was considered clinically insufficient, a cut-off value for prediction of hyperglycemia above 150 mg/dl on POD 1 was investigated. The cut-off value for preoperative HbA1c was chosen to be 5.9% on the ROC curve. Jämsen et al. reported similar results, and increased preoperative HbA1c was a risk factor for postoperative hyperglycemia in patients without DM [21]. HbA1c reflects BGLs over the past 2 or 3 months, and the complication rate following total joint arthroplasty was reported to increase linearly with higher HbA1c [22]. Patients with a high preoperative HbA1c, especially of 5.9% or more, may require careful perioperative glycemic control.

This study has some limitations. First, this study included a small number of patients, and BGLs on PODs 2 and 3 could not be studied in all cases. Since this study was conducted as an observational study, the BGL measurements on 2POD and 3POD were performed separately in the first and second half of the period, in consideration of patient and medical staff’s burden. Second, there was a 4-h difference in the start time between the first surgery in the morning and the surgery started in the afternoon. The difference in the start time of the surgery may have influenced the results of this study. Third, only fasting BGLs at the time of the examination were assessed and not random BGLs. Random BGL is likely to be even higher than fasting BGL. Although there were no cases of BGL exceeding 200 mg/dl in this study, the results might have been different if random BGLs were also assessed. Further research is needed to include both fasting and random BGLs.

## Conclusions

The postoperative BGL changes in patients without DM after unilateral primary TKA were highest at 7:00 AM on POD 1 and then decreased. Postoperative BGLs were significantly higher up to POD 2 compared to preoperative levels. Patients with an HbA1c of 5.9% or more had significantly higher BGLs than those with an HbA1c of under 5.9% on POD 1. Therefore, the optimal time period for BGL evaluation after TKA for patients without DM was considered to be from postoperative to 7:00 AM on POD 2. In addition, patients with preoperative HbA1c of 5.9% or more may require careful perioperative glycemic control.

## Data Availability

The datasets used and/or analysed during the current study are available from the corresponding author on reasonable request.

## Abbreviations

BGL: Blood glucose level
BMI: Body mass index
DM: Diabetes mellitus
DOS: Day of surgery
HbA1c: Hemoglobin A1c
POD: Postoperative day
ROC: Receiver operating characteristics
TKA: Total knee arthroplasty
